# 24 hour movement behaviours and the health and development of pre-school children from Zimbabwean settings: the SUNRISE pilot study

**DOI:** 10.17159/2078-516X/2021/v33i1a10864

**Published:** 2021-12-09

**Authors:** N Munambah, P Gretschel, F Muchirahondo, M Chiwaridzo, T Chikwanha, KE Kariippanon, KH Chong, PL Cross, CE Draper, AD Okely

**Affiliations:** 1 Rehabilitation Unit, Department of Primary Health Sciences, Faculty of Medicine, University of Zimbabwe, Zimbabwe; 2 Division of Health and Rehabilitation Sciences, Faculty of Health Sciences, University of Cape Town, South Africa; 3 Mental Health Unit, Department of Primary Health Sciences, Faculty of Medicine, University of Zimbabwe, Zimbabwe; 4 Early Start, School of Health and Society, Faculty of the Arts, Humanities and Social Sciences, University of Wollongong, NSW, Australia; 5 South African MRC Developmental Pathways for Health Research Unit, University of the Witwatersrand, South Africa; 6 Division of Exercise Science and Sports Medicine, UCT Research Centre for Health through Physical Activity, Lifestyle and Sport, University of Cape Town, South Africa

**Keywords:** early childhood development, physical activity, screen time, sleep

## Abstract

**Background:**

In 2019, the World Health Organization (WHO) released global guidelines for physical activity, sedentary behaviour and sleep for the early years. The International Study of Movement Behaviours in the Early Years, SUNRISE, aimed to assess the extent to which children aged three and four years meet the WHO global guidelines and its association with health and development.

**Objectives:**

To assess movement behaviours in pre-school children from low-income settings in Zimbabwe and to establish associations between these movement behaviours and adiposity, motor skills and executive function.

**Methods:**

Pre-school children/caregivers were recruited from two urban and two rural public schools respectively in Zimbabwe. The caregivers answered questions on the children’s physical activity, screen time, sedentary behaviour and sleep patterns. Children’s movement behaviours were objectively measured using accelerometers. Gross and fine motor skills and executive function were assessed using the Ages and Stages Questionnaire-3 and Early Years Toolbox, respectively. Focus group discussions were carried out with caregivers and teachers on the acceptability and feasibility of the study.

**Results:**

Eighty-one children participated in the study. The proportions of children meeting the guidelines were physical activity 92%, sedentary behaviour 70%, and sleep 86%, and all guidelines combined 24%. Boys and girls were similar (p>0.05 for all variables) for all executive function variables, but rural children had significantly lower inhibition scores (p=0.026) than urban children.

**Conclusion:**

The study adds to the growing literature on movement behaviours and associated risk factors in low-resourced settings. Further investigations of movement behaviours in this age group in Zimbabwe are recommended.

Promoting healthy movement behaviours (physical activity, sedentary behaviour and sleep) during a child’s early years (0 – 5 years) is fundamental to the development of each child, as it sets a foundation for facilitating and maintaining a healthy and active lifestyle throughout adulthood. ^[1]^ The World Health Organization’s (WHO) Ending Childhood Obesity (ECHO) Report highlighted the need to address 24-hour movement behaviours in early childhood for the prevention and management of obesity and non-communicable diseases (NCDs). ^[2]^ Following this, WHO developed global guidelines on physical activity, sedentary behaviour and sleep for children under five years of age which states that “pre-schoolers should be involved in at least 180 min of total physical activity (TPA) of which 60 min are spent in moderate to vigorous physical activity (MVPA) per day; (ii) screen time should be no more than one hour per day; and (iii) sleep duration between 10 to 13 h (including naps” ^[3]^. Canada, Australia and South Africa have also developed guidelines for physical activity, sedentary behaviour and sleep for the early years. ^[4–6]^

In Sub-Saharan Africa, rapid socio-cultural developments and urbanisation have resulted in a shift from traditional active lifestyles to more industrialised and sedentary lifestyles. ^[7]^ This has resulted in a decline in physical activity levels and an increase in sedentary behaviours mainly in adults, but with consequential effects on children. ^[7]^ According to the report card (Global Matrix 3.0) these risks could be high in Zimbabwe, where there is a double burden of malnutrition and associated childhood obesity*. ^[8]^ Despite the notable risks of overweight and obesity in children, few studies, most of which use self-reported measures, have been conducted in Zimbabwe. ^[9]^ There is a paucity of literature on physical inactivity and other movement behaviours as indicators for the prevention of NCDs among children in Zimbabwe. ^[8]^

The International Study of Movement Behaviours in the Early Years (SUNRISE), (https://sunrise-study.com) aims to determine the proportion of children sampled in participating countries who meet the WHO global guidelines for physical activity, sedentary behaviour and sleep for children under five years of age. Previously published SUNRISE studies in other countries have shown that a low proportion of children are meeting the WHO guidelines. ^[10]^ This paper on the Zimbabwean SUNRISE pilot study presents descriptive findings on movement behaviours in pre-school children from low-income urban and rural settings, and the associations between these movement behaviours and adiposity, motor skills and executive function (EF).

## Methods

### Ethical approval

A cross-sectional design was used in this study. Ethical clearance was obtained from the Medical Research Council of Zimbabwe (A/2385) and the Parirenyatwa Joint Research Ethics Committee (JREC 203/18). All public pre-schools in Zimbabwe are governed by the Ministry of Primary and Secondary Education, each of which is attached to a primary school. Thus, permission to access the pre-schools was sought from the Ministry of Primary and Secondary Education and the respective primary school authorities.

### Setting

This study took place at two rural and two urban public primary pre-schools registered by the Ministry of Primary and Secondary Education in Zimbabwe. Most rural areas do not have electricity, and children walk long distances to get to school. The urban areas are characterised by many cars, and electricity and water is readily available. ^[11]^ The study was conducted during the summer period (September 2018 – March 2019). Caregivers were invited to the school for a meeting where information about the study was provided and an appointment was set with each of the caregivers who expressed willingness to take part in the study. With the assistance from teachers, eligible children were invited to participate in the study. Written informed consent was obtained from parents/caregivers for the children to participate in the study.

### Participants

Children were eligible to participate if they were aged between four and five years, could wear an accelerometer, attended Early Childhood Development (ECD) education at the selected pre-schools, and if their primary caregivers were available to give consent and to respond to the questionnaire.

### Measures and procedures

Prior to data collection, four fieldworkers were trained in all aspects of the study’s protocol. Data collection from the children took place at each respective pre-school. The parent questionnaire was administered by trained field workers regarding the children either at home or at the child’s school.

### Anthropometry

The children’s height and weight were measured using a portable stadiometer (Leicester 214 Transportable Stadiometer; Seca, Germany) and a calibrated scale (Soehnle 7840 Mediscale Digital; Soehnle Industrial Solutions, Germany). All measurements were taken twice and the average was used for analysis. Height and weight were used to calculate the Body Mass Index (BMI). BMI and associated z-scores for BMI-for-age (BAZ), height-for-age (HAZ) and weight-for-age (WAZ) were computed using the WHO AnthroPlus software (http://www.who.int/growthref/tools/en/).

### Accelerometry

An ActivPal accelerometer was used to assess the 24-hour movement behaviours of the children. The ActivPALTM (PAL Technologies Ltd, Glasgow, UK), a small lightweight (15g) device, recorded the child’s sitting/lying, standing, and walking, transitions and step count. Each child wore the ActivPal on the right thigh continuously for 72 hours. Throughout the school day teachers also assisted with the monitoring and checking for compliance. The Accelerometry data was processed using the CREA algorithm of the ActivPal’s data processing software, PALbatch (Version 8.10.9.43, PAL Technologies Ltd., Glasgow, Scotland). Activity events were classified into seven main categories: total sitting/lying, standing, stepping and sleep time, number of bouts of sitting >30 min and time spent in motorised transport, based on thigh position and dynamic acceleration information. Furthermore, periods of sitting in motorised transport were identified (based on the presence of dynamic components in the acceleration signals from a seated event), and quantified periods of non-wear time (based on a measure of stillness of ≥60 min). Total sedentary behaviour was calculated by combining total sitting, and restrained sitting and lying times. Only participants with at least 20 h per day of wear data were included in the final analysis. ^[12]^

### Motor skills

In this study, motor skills included gross and fine motor skills. The Ages and Stages Questionnaire-3 (48 months, ASQ-3) was used to collect data on the gross and fine motors skills of the child. ^[13]^ Each child was asked to perform tasks as guided by the ASQ-3, and the research assistants observed and scored the performance of the child. The tasks for gross motor skills included catching a ball with both hands, throwing a ball overhead, and jumping forward over a distance of 50.8 cm from a standing position. The fine motor tasks assessed were: completing a wooden puzzle, cutting a piece of paper in half using a pair of scissors, unbuttoning one or more buttons of a shirt provided, drawing shapes on a piece of paper, and colouring in a shape using crayons.

### Executive function (EF) tests

In this study, EF tests were performed to assess the cognitive flexibility (shifting), control of behavioural urges and impulses (inhibition), and visual-spatial working memory of children using three short, iPad-based tools called the Early Years Toolbox (EYT). ^[14]^ The research assistant explained the games and participants practised for 10 minutes before recording was started. For each child, all three tasks were performed on an iPad using the same order. The tasks were performed in a quiet environment and took 20 minutes to complete per child. An exploratory factor analysis (EFA)-derived factor score (EF composite score) was computed for the three EYT tasks.

### Parent questionnaire

A parent questionnaire was developed and used to report basic sociodemographic data. The version of the parent questionnaire used in this study was the same as that used for all countries who participated in Phase 1 of the SUNRISE study. The parent questionnaire was translated into Shona and Ndebele (the most common local languages) and parents were asked to choose the language they were most comfortable in. Parents/caregivers were also asked to report on their child’s use of electronic media (to calculate sedentary screen time), sleep quality, and restrained sitting time.

### Focus group discussions

An interview guide was used to conduct four focus group discussions with purposively selected caregivers of children involved in the study. On average, each focus group discussion had nine caregivers and lasted between 45–60 minutes. Participants were asked about their experience with the data collection procedures, and the feasibility and acceptability of the selected instruments measuring movement behaviours.

### Statistical analysis

All analyses were performed using SPSS (v25.0, IBM Corp, Armonk, NY). Descriptive statistics were calculated using the mean and standard deviations (and median and interquartile range if not normally distributed) for continuous variables, and the frequency and percentage for categorical variables.

The differences between boys and girls and all the children from urban or rural areas were examined using independent t-tests or Mann-Whitney U tests for continuous variables, and Chi-square or Fisher’s Exact tests for categorical variables respectively. The correlations between accelerometer measures and BAZ, motor skills and executive functions were determined using the Spearman’s rank correlation coefficients. The differences in BAZ, motor skills and executive functions between meeting and not meeting movement behaviour guidelines (individual and integrated) were tested using Mann-Whitney U tests. Statistical significance was set at p<0.05. Data from the focus group discussions was transcribed verbatim and translated from the local languages to English. Thematic analysis was used to analyse the data. ^[15]^

## Results

The analytical sample was comprised of 81 children (n = 41 girls, 51%; n = 40 boys, 49%; n = 45 rural, 56%) from four pre-schools (Table 1). No significant differences were found between boys and girls for any anthropometric outcomes (all p > 0.05). According to the WHO cut-offs, 64% were in the normal BMI range, 12% were underweight, 15% overweight and 9% were obese. Compared with urban children, rural children had lower BMI, BAZ, and WAZ scores (p<0.0005).

Accelerometry, motor skills and executive function results are summarised in Table 2. Boys accrued a higher total stepping time (p=0.012) than girls. The differences between boys and girls across other movement behaviours were not statistically significant. Compared with urban children, those from rural areas had a higher total stepping time (p=0.025). There were no differences between boys and girls for any of the motor skill; however, urban children had significantly higher fine motor skill scores (p<0.0005) compared to their rural peers. For gross and fine motor skills, 86% and 42% of children were developmentally ‘on track’, respectively. Boys and girls were similar for all EF variables, but rural children had significantly lower inhibition scores (p=0.026) than urban children. More rural than urban children had fine motor skills categorised as at-risk or delayed.

Table 3 presents the results of the parent questionnaire. Differences between boys and girls for movement behaviours were small and not statistically significant. Compared with their urban peers, children in rural areas reported significantly more TPA (p = 0.002) and less screen time (p = 0.006), less sedentary time, (p <0.0001), and less time sitting in a vehicle on weekdays (p <0.0001) and weekends (p = 0.007).

**Fig. 1:**
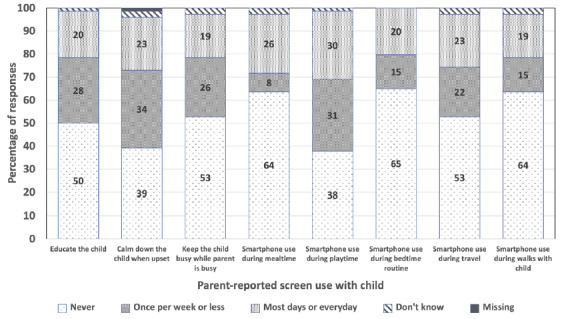
Frequency of parent-reported screen use with the child

Figure 1 shows the parent-reported screen time use with their child (also see supplementary data). More than half of the parents (55%) reported that their child used a screen before bed and 15% indicated that there was a screen in the room where the child sleeps. Parents of children in rural settings reported less screen usage before bed (p = 0.002) and fewer rural children (p = 0.005) had a screen in their bedroom compared with their urban counterparts. Seventy-two percent of parents reported that their child had consistent wake up times and 69% had consistent bedtimes. For 13% of the children, sleep quality was rated as ‘poor’ by parents, with significantly more rural than urban parents reporting poor sleep quality (p = 0.042). Only 13% of parents reported reading to their child every day, and in response to the question: During the past week, how many days did you or other household members read to this child? Sixty parents (74%) reported an average of 4.0±1.9 days.

**Fig. 2:**
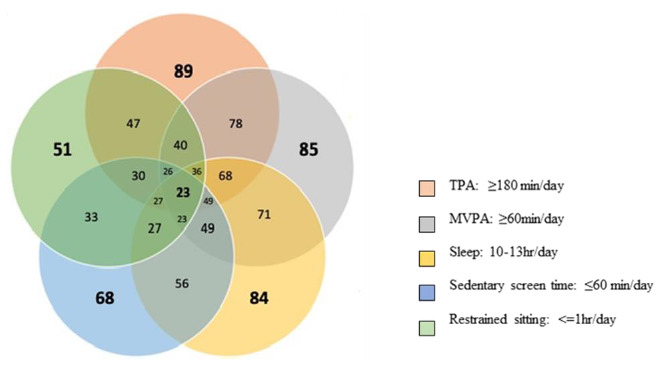
Venn diagram illustrating the proportion of children meeting all of the WHO 24-hour movement guidelines, meeting the guidelines individually and meeting combinations of the guidelines (n=71)

Figure 2 shows the proportion of children meeting the various components of the 24-hour movement guidelines. Nearly one quarter (23%) of children met all the components of the guidelines. Based on the parent questionnaire, most children met the guideline for TPA (89%), MVPA (85%) and sleep (84%). Compliance with the sedentary behaviour guidelines was considerably lower with 68% of parents reporting that, in the past week, their child had not spent ≤60 minutes per day engaging in sedentary screen time, and 51% of parents reported their child had not been restrained for >60 minutes at a time thus meeting these components of the sedentary behaviour guideline.

Associations between meeting the various guidelines and the health and developmental outcomes are reported in Table 4. Children who met the TPA and restrained sitting guidelines were found to have lower BAZ (p=0.026) but poorer working memory (p=0.016) compared to those who did not meet these guidelines. Children who met all components of the guidelines had lower BAZ (p=0.003) and displayed better gross motor skills (p=0.032), but had lower inhibition capabilities (p=0.018) compared with those who did not.

Positive associations were observed between time spent stepping and gross motor skills (r=0.34, p=0.012), time spent in sedentary behaviour and shifting (r=0.27, p=0.049), and time spent seated in motorised transportation and BAZ (r=0.31, p=0.019). No significant associations were observed for fine motor skills, working memory and inhibitions (See Table 5).

Two themes emerged from the focus group discussions. The first was ‘myths about the devices’ which explored the perception of caregivers and pre-school children on the use of the accelerometers. Despite providing detailed explanations about the devices and inviting questions, some caregivers thought that the devices had spiritual and or political connotations. They were hesitant about future implications and wanted further assurance of their safety. Caregivers also reported how children and adults were curious about the devices and were tempted to remove them so that they could explore the devices more closely. Teachers were helpful in explaining the purpose of the accelerometers and reassuring caregivers.

*‘I was very worried when I saw my child with that thing (Accelerometry device), I had to come here and ask the teacher, that is when I felt peace in my heart.’ (Participant 1)*

The second theme was ‘dynamics of consenting’ which reported on the cultural and contextual factors that affected consenting to the study and the use of self-report measures. The father, as the head of the family, was regarded as responsible for consenting and reporting about the child. However, at times fathers could not respond to specific questions about the child’s routine.

*‘It is correct that you have invited me to this meeting, Yes, I am the father but some of the information you are asking only the mother (of child) knows’. (Participant 2)*

Also, when a meeting was called, caregivers would send one person to represent the whole neighbourhood or an extended family relative; unfortunately, that person was not able to give consent for another person’s child.

## Discussion

This pilot study assessed movement behaviours in pre-school children from low-income settings in Zimbabwe. We found that it is appropriate to conduct studies of this nature in Zimbabwe. Through conducting this research, the SUNRISE team has learned how best (or how better) to conduct a bigger trial and/or other research where young children and parents in LMICs are required to contribute. More studies in this area will allow for population surveillance of movement behaviours and provide evidence to inform other interventions. ^[1]^ This is important in low-income countries where there are limited studies on movement behaviours in children. ^[4]^

This is the first study to examine the proportion of children meeting the WHO Global Guidelines for children under the age of five years in Zimbabwe. The only other such study undertaken was by Mushonga, Mujuru^[9]^, who used self-reported measures to collect data on factors associated with overweight/obesity among pre-school children. Although in this study the most knowledgeable parent needed to respond to the questionnaire, and this study confirmed a possible limitation with self-reported measures through a focus group discussion. It is important to highlight this possible limitation and design ways to increase the validity and reliability of data collected in future studies.

The proportion of children meeting all WHO Global Guidelines was higher than that reported among children from other lower to middle income countries (LMICs). For example, in China, where 15% met all three guidelines, in Guan, Zhang ^[10]^ and South Africa, 26% met all five guidelines ^[4]^. Noted variations in proportions could be attributed to the differences in accelerometer data collection (e.g. different types of physical activity monitors (e.g. ActivPAL vs Actigraph), and data reduction (e.g. different cut-points for light physical activity/MVPA) between studies would result in different estimates of physical activity. Similarly, the above reasons can be used to explain why children in Zimbabwe had a higher proportion of those who met the sleep guideline compared with China^10^ and South Africa. ^[4]^ However, we also found that a higher proportion of children from Zimbabwe met the screen time guideline compared with South Africa.^[4]^ Findings confirm the results of a study done in Zimbabwe by Mushonga, Mujuru ^[9]^ who reported that urban children spent more than three hours a day, especially on weekends, watching television or playing games. In contrast to participants in the South African SUNRISE study ^[4]^, most rural communities in Zimbabwe do not have access to electricity, hence children in rural settings were reported to have less screen usage before bed. Also, fewer rural children had a screen in their bedroom compared to the urban sample. The high number of children with access to the use of screens is worrying as research has shown that an increase in screen use is associated with obesity. ^[12]^

This study confirms previous findings that there is a significant proportion of children who are overweight and obese in Zimbabwe. ^[9]^ BMI and height were significantly lower in the rural population compared with the urban population. Further research on the growth and health of children against reports of undernutrition and stunting growth ^[8]^ in children in rural Zimbabwe might be required.

Most children who participated in this study performed well in the gross motor domain. The new ECD curriculum in Zimbabwe emphasises learning through play, and most pre-schools have a playground with swings and a specific time is aside for playing outside as part of the daily routines for children. ^[8]^ However, a comparison of rural vs urban children showed that rural children had a higher total stepping time than urban children. Limited play spaces coupled with readily accessible screen gadgets (for playing games) in urban areas could have predisposed urban children to having a lower total stepping time compared to rural children. ^[9]^

The strengths of this study include the use of well-established measures for this age group in low-income Zimbabwean settings, and the benefits of the collective expertise and experience of the SUNRISE global leadership group to inform the study design. The study also provided insights into aspects to consider when undertaking future studies on 24hr-movement behaviours of children from low resourced settings. More time should be invested in explaining the study to caregivers and the children as compliance to wearing the accelerometer is important in the reliability of the results. ^[10]^ Although children aged four-six years are very active and exploratory in nature, the tegaderm used to fix the accelerometer did not fall off. However, some children tried to remove the device out of curiosity in order to explore it further.

### Limitations

The main limitation of this pilot study was the small sample size and the study setting which was limited to one province in Zimbabwe. Thus findings cannot be generalised to other settings. This study used a cross-sectional design which precludes establishing causality.

## Conclusion

This pilot study contributes important initial findings on 24-hour movement behaviours of Zimbabwean pre-school children, and highlights that these behaviours require further attention in this age group. This is particularly important considering the growing risk of child obesity and high levels of screen time. Understanding how movement behaviours are associated with key outcomes, such as gross and fine motor skills as well as executive function in early childhood, is vital for setting children on their best trajectories for health and early learning. The study also provided insights into aspects to consider when undertaking future studies in this area.

## Supplementary Information



## Figures and Tables

**Table 1:** Sample age and anthropometric characteristics, by sex and setting

	Total (n=81)	Boys (n=40)	Girls (n=41)	p value‡	Rural (n=45)	Urban (n=36)	p value§
**Age (y)**	5.2 ± 0.5	5.2 ± 0.4	5.1 ± 0.5	0.290	5.1 ± 0.5	5.2 ± 0.4	0.097
**Height (cm)**	106.8 ± 4.4	107.3 ± 4.7	106.3 ± 4.2	0.309	106.2 ± 4.6	107.5 ± 4.2	0.193
**Weight (kg)**	17.5 ± 2.8 17.0 (16.0 – 19.0)	17.9 ± 2.9 17.8 (16.0 – 20.0)	17.0 ± 2.7 17.0 (15.5 – 19.0)	0.165	15.8 ± 2.0 16.0 (14.0 – 17.0)	19.5 ± 2.5 19.0 (18.0 – 20.4)	<0.0005*
**BMI (kg.m** **^−2^** **)**	15.3 ± 2.3 14.9 (13.9 – 16.6)	15.6 ± 2.6 15.0 (14.0 – 17.0)	15.0 ± 1.9 14.7 (13.6 – 16.5)	0.377	14.1 ± 1.5 14.2 (13.0 – 14.9)	16.9 ± 2.1 16.6 (15.2 – 17.9)	<0.0005*
**HAZ**	−0.76 ± 1.10 (−1.52 – −0.08)	−0.79 ± 1.24 −0.74 (−1.76 – −0.14)	−0.72 ± 0.96 −1.02 (−1.28 – 0.00)	0.643	−0.78 ± 1.18 −0.89 (−1.54 – −0.05)	−0.72 ± 1.02 −0.92 (−1.51 – −0.17)	0.890
**WAZ**	−0.55 ± 1.16	−0.46 ± 1.22	−0.65 ± 1.10	0.465	−1.18 ± 0.93	0.22 ± 0.92	<0.0005*
**BAZ**	−0.15 ± 1.60	0.04 ± 1.84	−0.32 ± 1.32	0.313	−1.03 ± 1.27	0.97 ± 1.23	<0.0005*

Data are presented as mean ± SD for normally distributed data; not normally distributed data also includes median (25^th^ – 75^th^ percentile).

* indicates significance at p<0.0005;

‡ p value for comparison by sex;

§ p value for comparison by setting. BMI, body mass index; HAZ, height-for-age z-score; WAZ, weight-for-age z-score; BAZ, BMI-for-age z-score.

**Table 2:** Accelerometer measures, motor skills and executive function results, by sex and setting

Accelerometer measures	Total (n=58)	Boys (n=30)	Girls (n=28)	p value‡	Rural (n=36)	Urban (n=22)	p value§
**Total stepping (min/day)**	228.2 ± 43.1	241.7 ± 38.7	213.7 ± 43.6	0.012*	238.0 ± 42.6	212.1 ±39.9	0.025*
**Total standing (min/day)**	258.5 ± 41.0	252.3 ± 39.8	265.1 ± 41.9	0.235	255.3 ± 41.6	263.6 ± 40.2	0.459
**Total sitting (min/day)**	323.5 ± 62.4	324.2 ± 60.7	322.8 ± 65.3	0.930	312.9 ± 56.0	340.9 ± 69.5	0.097
**Lying (min/day)**	23.2 ± 50.8 0.0 (0.0 – 31.8)	20.4 ± 44.6 0.0 (0.0 – 8.0)	26.3 ± 57.4 0.0 (0.0 – 36.1)	0.707	20.4 ± 51.6 0.0 (0.0 – 0.0)	27.9 ± 50.3 0.0 (0.0 – 41.7)	0.405
**Total SB (min/day)**	385.2 ± 84.9	379.7.1 ± 91.0	391.3 ± 78.8	0.370	385.2 ± 81.4	385.0 ± 92.0	0.630
**Bouts >30 min**	0.26 ± 0.61 0.00 (0.00 – 0.00)	0.37 ± 0.72 0.00 (0.00 – 1.00)	0.14 ± 0.45 0.00 (0.00 – 0.00)	0.129	0.17 ± 0.45 0.00 (0.00 – 0.00)	0.41 ± 0.80 0.00 (0.00 – 1.00)	0.192
**Motorised transportation (min/day)**	39.7 ± 32.4 30.8 (16.8 – 53.1)	41.5 ± 33.9 33.2 (16.8 – 55.0)	37.8 ± 31.2 29.8 (16.5 – 51.9)	0.663	41.1 ± 34.6 31.0 (16.7 – 59.0)	37.5 ± 29.0 30.8 (18.9 – 51.9)	0.873
**Sleep (min/day)**	605.0 ± 65.1	606.8 ± 74.0	603.2 ± 55.3	0.835	586.5 ± 62.4	585.3 ± 63.5	0.070

**Motor skills**	**Total (n=78)**	**Boys (n=39)**	**Girls (n=39)**	**p value** ‡	**Rural (n=42)**	**Urban (n=36)**	**p value** §

**Gross motor skills**	55.9 ± 7.4 60.0 (54.0 – 60.0)	55.3 ± 8.7 60.0 (54.0 – 60.0)	56.6 ± 5.8 60.0 (54.0 – 60.0)	0.811	56.7 ± 7.2 60.0 (58.5 – 60.0)	55.0 ± 7.6 60.0 (49.5 – 60.0)	0.173
**Fine motor skills**	40.5 ± 12.7	38.9 ± 12.5	42.2 ± 12.7	0.247	33.5 ± 10.5	48.8 ± 9.7	<0.0005**

**Executive function**	**Total (n=78)**	**Boys (n=39)**	**Girls (n=39)**	**p value** ‡	**Rural (n=42)**	**Urban (n=36)**	**p value** §

**Working memory** †	1.09 ± 1.01 1.00 (0.00 – 2.00)	1.14 ± 1.17 0.83 (0.0 – 2.08)	1.03 ± 0.81 1.00 (0.08 – 1.83)	0.930	0.90 ± 0.83 1.00 (0.00 – 1.33)	1.30 ± 1.16 1.50 (0.00 – 2.08)	0.144
**Inhibition** †	0.59 ± 0.22	0.57 ± 0.21	0.60 ± 0.22	0.569	0.54 ± 0.20	0.65 ± 0.22	0.026*
**Shifting** †	5.34 ± 4.49 4.00 (0.00 – 9.00)	5.50 ± 4.41 5.00 (1.00 – 9.00)	5.18 ± 4.61 4.00 (0.00 – 9.00)	0.689	5.51 ± 4.23 7.00 (1.50 – 9.00)	5.12 ± 4.85 4.00 (0.00 – 10.00)	0.712

Data are presented as mean ± SD for normally distributed data; not normally distributed data also includes median (25^th^ – 75^th^ percentile).

* indicates significance at p<0.05;

** indicates significance at p<0.0005;

‡ p value for comparison by sex;

§ p value for comparison by setting. SB, sedentary behaviour (sitting, restrained sitting and lying combined); EF, executive function;

† indicates missing data for working memory (n=4; 1 boy and 3 girls; 2 from rural setting and 2 from urban setting), inhibition (n=2; 1 boy and 1 girl; 1 from rural setting and 1 from urban setting), shifting (n=4; 3 boys and 1 girls; 1 from rural setting and 3 from urban setting).

**Table 3:** Parent questionnaire (continuous variables), by sex and setting

	Total (n=74)	Boys (n=35)	Girls (n=39)	p value‡	Rural (n=44)	Urban (n=30)	p value§
**TPA (hr/d)**	6.4 ± 2.3 8.0 (4.0 – 8.1)	6.6 ± 2.3 8.0 (4.5 – 8.0)	6.2 ± 2.4 7.3 (4.0 – 8.3)	0.646	7.1 ± 1.9 8.0 (6.3 – 8.3)	5.3 ± 2.5 5.5 (2.9 – 8.0)	0.002*
**MVPA (hr/d)**	2.7 ± 2.0 2.3 (1.0 – 4.0)	2.6 ± 2.0 2.2 (1.0 – 4.0)	2.8 ± 2.0 2.3 (1.0 – 4.0)	0.572	2.8± 2.2 2.1 (1.0 – 3.9)	2.7 ± 1.7 2.6 (1.0 – 4.0)	0.847
**Screen time (hr/d)** †	1.6 ± 1.6 1.0 (0.3 – 2.4)	1.6 ± 1.8 1.0 (0.0 – 2.5)	1.6 ± 1.5 1.0 (0.3 – 2.4)	0.697	1.1 ± 1.3 0.8 (0.0 – 2.0)	2.2 ± 1.9 1.5 (0.8– 4.0)	0.006*
**Sleep (hr/d)** †	10.3 ± 1.5	10.5 ± 1.3	10.1 ±1.6	0.247	10.6 ± 1.6	10.0 ± 1.1	0.056
**Time spent sitting (hr/d)** †	1.8 ± 2.2 1.0 (0.4 – 2.2)	1.3 ± 1.3 1.0 (0.3 – 2.0)	2.3 ± 2.7 1.0 (0.5 – 3.3)	0.211	1.1 ± 2.0 0.5 (0.3 – 1.1)	2.8 ± 2.1 2.0 (1.0 – 5.0)	<0.0001**
**Time spent sitting in a vehicle (weekdays, hr/d)**	0.8 ± 1.8 0.0 (0.0 – 1.0)	1.1 ± 2.4 0.0 (0.0 – 1.0)	0.6 ± 1.2 0.0 (0.0 – 1.0)	0.686	0.2 ± 0.4 0.0 (0.0 – 0.1)	1.8 ± 2.6 0.8 (0.0 – 2.0)	<0.0001**
**Time spent sitting in a vehicle (weekends, hr/d)**	0.5 ± 0.7 0.0 (0.0 – 1.0)	0.5 ± 0.7 0.0 (0.0 – 1.0)	0.5 ± 0.9 0.0 (0.0 – 1.0)	0.842	0.4 ± 0.8 0.0 (0.0 – 0.5)	0.7 ± 0.6 0.6 (0.0 – 1.0)	0.007*

Data are presented as mean ± SD and median (25th – 75th percentile).

* indicates significance at p<0.01;

** indicates significance at p<0.0001;

‡ p value for comparison by sex;

§ p value for comparison by setting. TPA, total physical activity; MVPA, moderate-vigorous intensity physical activity;

† indicates missing data for screen time (n=1; 1 girl from urban setting), sleep (n=2; 2 girls from urban setting), time spent sitting (n=1; 1 boy from urban setting).

**Table 4:** Comparison of BAZ, gross motor skills, fine motor skills, executive functions (working memory, inhibition, shifting) by meeting/not meeting individual and integrated movement behaviour guidelines

	TPA		MVPA		SST		Sleep duration		Restrained sitting		All five guidelines	

	Yes (n=65)	No (n=6)	Yes (n=62)	No (n=9)	Yes (n=37)	No (n=34)	Yes (n=50)	No (n=21)	Yes (n=61)	No (n=10)	Yes (n=17)	No (n=54)

**BAZ**												
Median	−0.43	0.75	−0.42	0.17	−0.55	−0.13	−0.39	−0.20	−0.43	0.32	−1.25	−0.21
IQR	−1.58–0.58	0.35–1.85	−1.31–0.69	−2.21–1.06	−1.61–0.32	−0.74–1.03	−1.53–0.80	−1.22–0.74	−1.60–0.63	−0.55–1.73	−1.84–−0.37	−0.77–1.02
MR	34.37	53.67	35.63	38.56	31.86	40.86	35.24	37.81	34.24	46.75	23.12	40.06

**p-value**	0.026*		0.691		0.078		0.632		0.076		0.003**	

**Gross motor skills**												
Median	60.0	57.0	60.0	60.0	60.0	60.0	60.0	60.0	60.0	60.0	60.0	60.0
IQR	58.5–60.0	54.0–60.0	54.0–60.0	54.0–60.0	60.0–60.0	54.0–60.0	60.0–60.0	54.0–60.0	54.0–60.0	58.5–60.0	60.0–60.0	54.0–60.0
MR	35.03	29.00	34.71	33.11	37.32	31.33	35.30	32.71	34.06	37.05	41.69	32.29

**p-value**	0.492		0.770		0.108		0.521		0.569		0.032*	

**Fine motor skills**												
Median	40.0	42.5	40.0	35.0	40.0	40.0	40.0	45.0	40.0	42.5	37.5	40.0
IQR	30.0–55.0	40.0–56.2	30.0–55.0	30.0–52.5	31.25–50.0	25.0–55.0	30.0–50.0	32.5–55.0	30.0–55.0	35.0–55.0	30.0–45.0	30.0–55.0
MR	33.59	43.92	35.06	30.83	34.35	34.67	32.78	38.36	33.76	38.80	30.53	35.72

**p-value**	0.228		0.547		0.946		0.279		0.453		0.355	

**Working memory**												
Median	1.00	1.00	1.00	1.33	1.00	1.00	1.00	1.00	1.00	2.00	0.67	1.00
IQR	0.00–2.00	0.00–2.00	0.00–1.92	0.33–2.17	0.00–1.92	0.00–2.00	0.00–2.00	0.00–1.33	0.00–1.67	0.75–2.41	0.00–1.67	0.00–2.00
MR	33.15	31.50	32.06	38.83	31.83	34.45	34.24	30.00	30.65	45.95	29.32	34.30

**p-value**	0.851		0.309		0.572		0.402		0.016*		0.341	

**Inhibition**												
Median	0.60	0.46	0.59	0.70	0.57	0.62	0.58	0.62	0.59	0.63	0.51	0.63
IQR	0.47–0.75	0.30––0.77	0.46–0.72	0.31–0.82	0.39–0.71	0.48–0.76	0.46–0.71	0.43–0.81	0.42–0.73	0.48–0.92	0.30–0.64	0.47–0.81
MR	34.66	27.33	33.60	36.94	31.62	36.76	33.01	36.33	32.79	40.90	24.35	37.28

**p-value**	0.395		0.650		0.282		0.524		0.225		0.018*	

**Shifting**												
Median	7.00	2.00	5.50	4.00	7.00	4.00	5.50	4.00	5.50	4.00	8.00	4.00
IQR	1.00–9.00	0.00–9.00	0.25–9.00	1.00–9.00	1.00–9.00	0.75–9.25	0.75–10.00	1.00–9.00	1.00–9.00	1.00–11.50	0.00–9.00	1.00–9.75
MR	33.69	26.17	33.17	31.94	33.24	32.72	33.82	31.03	32.72	34.72	33.44	32.84

**p-value**	0.368		0.855		0.910		0.910		0.766		0.910	

* indicates significance at p<0.05;

** indicates significance at p<0.0005.

“Yes” indicates the participants that met the guidelines and “No” indicates the participants that did not meet the guidelines. MR, mean ranks; IQR, interquartile range (presented as 25^th^ – 75^th^ percentile); BAZ, BMI-for-age z score; TPA, total physical activity; MVPA, moderate-vigorous intensity physical activity; SST, sedentary screen time.

**Table 5:** Correlations between accelerometer measures and BAZ, gross motor skills, fine motor skills and executive functions (working memory, inhibition, shifting)

Guideline	BAZ (n=58)		Gross motor skills (n=55)		Fine motor skills (n=55)		Working memory (n=52)		Inhibitions (n=53)		Shifting (n=53)	

	r	p-value	r	p-value	r	p-value	r	p-value	r	p-value	r	p-value

Total stepping (min/day)	−0.07	0.589	0.34	0.012*	−0.09	0.496	0.09	0.550	0.13	0.372	0.12	0.404
Total standing (min/day)	−0.04	0.749	−0.17	0.226	−0.11	0.411	0.25	0.080	0.10	0.500	−0.15	0.283
Total sitting (min/day)	0.24	0.070	−0.18	0.188	0.15	0.263	−0.08	0.562	−0.218	0.117	0.110	0.432
Lying (min/day)	−0.11	0.431	−0.18	0.193	0.22	0.113	−0.11	0.431	0.03	0.851	0.27	0.049*
Total SB (min/day)	−0.04	0.767	−0.07	0.602	0.09	0.498	0.12	0.410	−0.17	0.227	−0.01	0.923
Restricted sitting (min/day)	0.31	0.019*	0.04	0.796	−0.03	0.857	−0.25	0.073	−0.01	0.954	0.27	0.055
Sleep (min/day)	−0.06	0.633	0.12	0.388	−0.19	0.160	−0.15	0.278	0.05	0.738	−0.06	0.651

* indicates significance at p<0.01.

BAZ, BMI-for-age z score; SB, sedentary behaviours; r, Spearman’s correlation coefficients.

## Footnotes

* Undernutrition is synonymous with malnutrition which denotes insufficient intake of energy and nutrients to meet an individual’s needs to maintain good health. Forms of undernutrition include wasting, stunting, underweight and deficiencies in vitamins and minerals.
