# Supplementary material for: 24 hour movement behaviours and the health and development of pre-school children from Zimbabwean settings: the SUNRISE pilot study
Source: S Afr J Sports Med. 2021 Dec 9;33(1):v33i1a10864. doi: 10.17159/2078-516X/2021/v33i1a10864 (PMC9924604; doi:10.17159/2078-516X/2021/v33i1a10864)
Supplement: Supplementary file 1 [file 2078-516X-33-v33i1a10864-s001.pdf]

Supplementary data:

**Variables**	**N valid response**	**n (%)**	**Urban**	**Rural**	**p-value**
Gross motor skills[a]	78				0.551
‘Requires follow-up and further assessment/action’		3 (3.8)	1	2	
‘Developing on schedule but may benefit from extra practice in some of the skills’		6 (7.7)	4	2	
‘Developing on schedule’		69 (88.5)	31	38	
Fine motor skills[b]	78				**<0.0001*** **
‘Requires follow-up and further assessment/action’		12 (15.4)	0	12	
‘Developing on schedule but may benefit from extra practice in some of the skills’		33 (42.3)	11	22	
‘Developing on schedule’		33 (42.3)	25	8	
Screen usage before bed[b]	74	41 (55.4)	23 (76.7)	18 (40.9)	**0.002*** **
Presence of screen in bedroom[a]	74	11 (14.9)	9 (30.0)	2 (4.5)	**0.005*** **
Consistent bed time[b]	74	51 (68.9)	18 (60.0)	33 (75.0)	0.171
Consistent wake up time[b]	74	53 (71.6)	21 (70.0)	32 (72.7)	0.798
Poor quality (rating 3 or below)[a]	74	10 (13.5)	1 (3.3)	9 (20.5)	**0.042***
Frequency of reading to child - Every day[a]	60	10 (13.5)	2 (8.3)	8 (22.2)	0.289
Restrained sitting for more than 1 hour[a]	74	11 (14.9)	7 (23.3)	4 (9.1)	0.108

Differences between urban and rural setting were analysed using: [a]Fisher’s Exact test; [b]Chi-Square test.
Bold value indicates statistically significant association at *p<0.05 and ***p<0.001.
